# Revolutionizing Diabetes Diagnosis: Machine Learning Techniques Unleashed

**DOI:** 10.3390/healthcare11212864

**Published:** 2023-10-31

**Authors:** Zain Shaukat, Wisal Zafar, Waqas Ahmad, Ihtisham Ul Haq, Ghassan Husnain, Mosleh Hmoud Al-Adhaileh, Yazeed Yasin Ghadi, Abdulmohsen Algarni

**Affiliations:** 1 Department of Computer Science, Iqra National University Peshawar, Peshawar 25100, Pakistan; 2 Department of Mechatronics Engineering, UET Peshawar, Peshawar 25000, Pakistan; 3 eLearning and Distance Education, King Faisal University, Al-Ahsa 7057, Saudi Arabia; 4 Department of Computer Science, Al Ain University, Abu Dhabi P.O. Box 112612, United Arab Emirates; yazeed.ghadi@aau.ac.ae; 5 Department of Computer Science, King Khalid University, Abha 61421, Saudi Arabia

**Keywords:** PIMA diabetes dataset, WEKA, Matthew’s correlation coefficient, Python, accuracy, machine learning algorithms, diabetes

## Abstract

The intricate and multifaceted nature of diabetes disrupts the body’s crucial glucose processing mechanism, which serves as a fundamental energy source for the cells. This research aims to predict the occurrence of diabetes in individuals by harnessing the power of machine learning algorithms, utilizing the PIMA diabetes dataset. The selected algorithms employed in this study encompass Decision Tree, K-Nearest Neighbor, Random Forest, Logistic Regression, and Support Vector Machine. To execute the experiments, two software tools, namely Waikato Environment for Knowledge Analysis (WEKA) version 3.8.1 and Python version 3.10, were utilized. To evaluate the performance of the algorithms, several metrics were employed, including true positive rate, false positive rate, precision, recall, F-measure, Matthew’s correlation coefficient, receiver operating characteristic area, and precision–recall curves area. Furthermore, various errors such as Mean Absolute Error, Root Mean Squared Error, Relative Absolute Error, and Root Relative Squared Error were examined to assess the accuracy of the models. Upon conducting the experiments, it was observed that Logistic Regression outperformed the other techniques, exhibiting the highest precision of 81 percent using Python and 80.43 percent using WEKA. These findings shed light on the efficacy of machine learning in predicting diabetes and highlight the potential of Logistic Regression as a valuable tool in this domain.

## 1. Introduction

Diabetes is a complicated and complex medical illness that has an impact on how the body utilizes glucose, a form of sugar that is the body’s primary fuel source. When our body is unable to create insulin or is unable to use the insulin that is produced by our bodies, diabetes results. According to statistics from 2017, 425 million people worldwide have diabetes. Diabetes causes 2 to 5 million deaths worldwide each year. By 2045, it is projected to grow to 629 million [1,2]. Type 1 diabetes, Type 2 diabetes, and gestational diabetes are the three primary subtypes of diabetes, which are categorized according to their underlying causes and physical effects.

Type 1 IDDM (Insulin-Dependent Diabetes Mellitus), also known as Type 1 diabetes, occurs when the body is unable to produce sufficient insulin, leading to a dependence on insulin injections for patients with this condition.

Type 2 is otherwise called Non-Insulin-Subordinate Diabetes Mellitus (NIDDM). This kind of diabetes is caused when the body’s cells cannot utilize insulin appropriately.

Type 3 is called gestational diabetes, which is the aftereffect of an expansion in glucose levels during incubation. These pregnant women have not been diagnosed with any kind of diabetes before. There are long-term complications associated with diabetes. Diabetes can likewise prompt a higher risk of different medical conditions.

Data mining is the process of finding insights into data, which helps in the prediction of an event before it happens. This technique is used to find some useful information from the raw data provided. If these techniques are accurately analyzed and applied to datasets, they can predict events when appropriate algorithms are used on training and testing datasets. In the previous paper we studied and reviewed, the diabetes prediction system required a small dataset [3], whereas the Pima Indians Diabetes Dataset (PIDD), which is accessible online in the UCI Machine Learning Repository [4], consists of 786 instances, and a total of 9 of these attributes are used in this research. Different machine learning algorithms, such as Logistic Regression (LG), Support Vector Machines (SVM), Decision Tree (DT), Random Forest (RF), and K-Nearest Neighbor (KNN) classification algorithms, were performed against the Pima Indians Diabetes Dataset (PIDD). We examined all of the above algorithms and observed that each algorithm gives a result with different accuracies. Among all of the above algorithms, we picked out the ones which gave us the most accurate results. Decision Tree and Logistic Regression were therefore chosen, since they had the highest accuracy rates of 81% and 80%, respectively. The diabetic researcher is therefore curious to observe the most recent studies utilizing various machine learning approaches. Diabetes is one of the main priorities of medical research, which generates a lot of data due to the substantial social impact of this particular disease. Research is a crucial strategy that can be used to develop knowledge to efficiently utilize sizable amounts of diabetes-related data.

The current study contributes to the existing literature by conducting a comprehensive analysis of six classification algorithms for diabetes prediction, shedding light on their relative performance and identifying Logistic Regression as the most precise approach. This study employs a diverse set of performance metrics and multiple software tools, demonstrating methodological versatility. Furthermore, it outlines plans for the development of web-based and Android applications for diabetes prediction and management, emphasizing the practical implications of the research. The commitment to ongoing research and algorithm refinement adds to the literature by promoting continuous innovation in healthcare-related machine learning applications, ultimately enhancing our understanding of the potential of machine learning in addressing diabetes and other healthcare challenges.

## 2. Related Works

In the Diabetes Using Classification Algorithms (PDCA) research paper, Deepti Sisodia et al. (2018) compared only three algorithms, namely Decision Tree, Naïve Bayes, and Support Vector Machine (SVM) in experiments performed by using Pima Indians Diabetes Dataset (PIDD). By applying different classification algorithms, the best and highest accuracy is from Naïve Bayes of at 76.30% [5].

In the Predicting Diabetes Mellitus using Data Mining Techniques (PDMDMT) research paper by J. Steffi, Dr. R. Balasubramanian et al. (2018), the authors compared five algorithms, namely Artificial Neural Network (ANN), Naïve Bayes, Support Vector Machine (SVM), Logistic Regression, and C5.0 [6].

As proven by the Improved J48 Classification Algorithms for the Prediction of the Diabetes research paper, Gaganjot Kaur and Amit Chhabra et al. (July 2014) used the J48 Decision Tree algorithm to improve the accuracy of the J48 algorithm and the C4.5. These algorithms were used with an actual accuracy of 73.8281% and the accuracy of the proposed algorithm is 99.87% [7].

Madhusmita Rout, Amandeep Kaur et al. (2019) applied SVM, Naïve Bayes, Decision Tree, KNN, and Logistic Regression techniques to the proposed prediction model and made an accurate comparison of those algorithms. Experimental results show that the Logistic algorithm had an accuracy of 82.35%, which has proven to be better than the other applied algorithms [8].

Varma, K. M., and Panda, B. S. et al. (2019) showed how the data mining classification algorithms C5.0, Logistic Regression, Naïve Bayes, Support Vector Machine, and Artificial Neural Networks are utilized to demonstrate genuine diabetes mellitus predictions. Thereafter, a near investigation is made between them, utilizing metric system. As a result of the research work, C5.0 and Logistic Regression were equally good based on their accuracy measurements of 74.63% and 74.67%, respectively [9].

Wu, H., Yang, S., Huang, Z., He, J., and Wang, X. et al. (2018) used the PIDD dataset with the K-Means and Logistic Regression algorithms to perform the task, which has the best accuracy of 95.42% [10].

O. Dr. O., S. Dr. K., and B. Ramudu et al. (2020) “Used machine learning Techniques for diabetes prediction”. The study utilized several regression algorithms, including SVM, KNN, and Artificial Neural Networks (ANN), to analyze the PIMA diabetes dataset. The research was conducted using R-Studio software 4.0.2 version, and Logistic Regression was found to have the highest accuracy at 78% [11].

Das H., Naik, B., and Behera, H. et al. (2018) investigated diabetes forecasts utilizing different order calculations, for example, J48 and Bayesian naivety, to analyze the infection rapidly. The proposed model aids patients and clinicians in saving time by creating reports rapidly from an information store. It was assessed utilizing WEKA and MATLAB programming. Further investigation can be conducted with a mixture of information-digging procedures for accurate and precise outcomes [12].

Abdulhadi, N., and Al-mousa, A. et al. (2021) used different machine learning algorithms like Logistic Regression Classifier, Linear Discriminant Analysis (LDA), Linear Support Vector Machine, SVC (Support Vector Classifier) with Polynomial Kernel, Random Forest Classifier, and Voting Classifier to evaluate the different accuracies. The best ML algorithm among them is the Random Forest classifier, which achieves 82% accuracy on the PIMA diabetes dataset [13].

Based on the study by Chou, C.-Y., Hsu, D.-Y., Chou, C.-H. et al. (2023) diabetes prediction was performed and evaluated by applying various machine learning algorithms, including Logistic Regression, Binary Neural Network, Decision Forest, and Decision Tree to evaluate model performance, accuracy, precision, recall, and the F-measure. The two-level augmented Decision Tree was the most efficient model, outperforming all other models on the PIMA diabetes dataset with an augmented patient ID under curve result of 0.991 [14]. A comparison of our proposed study with previous literature studies is mentioned and illustrated in Table 1 below.

## 3. Model and Classification Algorithms

We encourage research data to be archived in data repositories wherever possible.

### 3.1. Dataset Description

We utilized the PIMA diabetes dataset from the UCI Machine Learning Repository for this study [4]. The 768 illustrations, 8 characteristics, and 1 class attribute that carries the values tested positive (1) and tested negative (0) make up this dataset [5]. Two toolboxes, specifically the WEKA (Waikato Environment for Knowledge Analysis) device developed by the University of Waikato, New Zealand, which contains apparatuses for data pre-processing, characterization, and relapse [20,21], are used in this paper to perform and really examine the exactness and execution of various order calculations. Table 2 depicts the datasets, attributes, minimum, maximum, mean, and standard deviation (SD).

#### 3.1.1. Simple Size

The dataset consists of 768 observations (rows), making it a moderate dataset for a machine learning study.

#### 3.1.2. Features

There are eight attributes (columns) in the dataset, including:

- Number of pregnancies;
- Plasma glucose concentration;
- Diastolic blood pressure;
- Triceps skinfold thickness;
- 2 h serum insulin;
- Body mass index (BMI);
- Diabetes pedigree function;
- Age.

#### 3.1.3. Target Variable

The binary variable being targeted indicates whether diabetes is detected or not. Generally, a value of 1 indicates the presence of diabetes, while a value of 0 indicates its absence.

#### 3.1.4. Sex Distribution

There is no significant gender distribution in the dataset. It mainly focuses on diabetes status and health-related features. Therefore, the dataset itself does not contain information on gender distribution.

#### 3.1.5. Diabetes Prevalence

The dataset contains details of the diabetes status of 768 sample members, including whether they have diabetes or not. By multiplying the number of people with diabetes (target variable = 1) and dividing it by the total number of observations (768), it is possible to estimate the prevalence of diabetes in the dataset.

#### 3.1.6. Missing Values

Datasets may contain missing values, which can affect data analysis and modeling. Data preprocessing steps may be required to handle missing data.

### 3.2. Detailed Accuracy

Generally, we know that the prediction process consists of our main attributes, which are called true positive (TP), true negative (TN), false positive (FP), and false negative (FN) [6] and are used to calculate the precision, recall, MCC, F-measure score, true positive rate (TPR), true negative rate (TNR), false positive rate (FPR), false negative rate (FNR), and accuracy.

#### 3.2.1. Precision

A classification model’s precision is a metric that assesses its capacity to correctly identify only the pertinent data points [22]. Mathematically, precision can be computed using the formula below:

(1) $$Precision=\frac{TP}{TP+FP}$$

By using Equation (1), we can calculate the precision value [22].

#### 3.2.2. Recall

The recall is a metric that evaluates the ability of a model to identify all the relevant cases within a dataset [14]. Mathematically, recall can be computed using the formula below:

(2) $$Recall=\frac{TP}{TP+FN}\;$$

By using Equation (2), we can calculate the recall value [14].

#### 3.2.3. Mathews Correlation Coefficient

The Mathews correlation coefficient (MCC) is more robust statistical measure than others, as it produces a higher score when the model achieves good outcomes across all four classes of the confusion matrix (true positive, false negative, true negative, and false positive) relative to the size of the positive and negative components in the dataset [22]. Mathematically, the MCC can be calculated by using the formula below:

(3) $$MCC=\frac{\left(\left(TN*TP\right)-\left(FN*FP\right)\right)}{\sqrt{\left(\left(FP+TP\right)*\left(FP+TN\right)*\;\left(FP+TP\right)*\left(FP+TN\right)\right)}}$$

By using Equation (3), we can calculate the MCC value [22].

#### 3.2.4. True Positive Rate

A true positive is an outcome where the model correctly predicts the positive value. Mathematically, the true positive can be calculated by the formula below:

(4) $$TPR=\frac{TP}{FN+TP}$$

By using Equation (4), we can calculate the TPR value.

#### 3.2.5. True Negative Rate

In a classification problem, a true negative refers to an outcome where the model correctly predicts the negative class. Mathematically, the true negative can be calculated by using the formula below:

(5) $$TNR=\frac{TN}{TN+FP}$$

By using Equation (5), we can calculate the TNR value.

#### 3.2.6. False Positive Rate

In a classification problem, a false positive is an outcome where the model incorrectly predicts the positive class. Mathematically, the false positive rate can be calculated by using the formula below:

(6) $$FPR=\frac{FP}{TN+FP}$$

By using Equation (6), we can calculate the FPR value.

#### 3.2.7. False Negative Rate

A false negative occurs when the model predicts a negative class. False negatives are a type of error in which the model fails to detect a true positive instance. Mathematically, the false negative can be calculated by using the formula below:

(7) $$FNR=\frac{FN}{TP+FN}$$

By using Equation (7), we can calculate the FNR value.

#### 3.2.8. Mean Absolute Error

The formula calculates the sum of the absolute differences between each predicted value and its corresponding true value and then divides by the total number of predictions to obtain the average error. The lower the MAE value, the better the predictive model is performing. Mathematically, the MAE can be calculated by using the formula below:

(8) $$MAE=\frac{1}{2}\overset{n}{\underset{i=0}{\sum }}\left|{\hat{y}}_{i}-y_{i}\right|\;$$

By using Equation (8), we can calculate the MAE value.

#### 3.2.9. Root Mean Squared Error

RMSE is the standard deviation of errors that occur when predicted on a dataset. This is similar to MSE (Main Squared Error), but the root of the value is considered when determining the accuracy of the model. Mathematically, the RMSE can be calculated by using the formula below [23]:

(9) $$MSE=\sqrt{\frac{1}{2}\overset{n}{\underset{i=0}{\sum }}{\left(Y_{i}-{\hat{Y}}_{i}\right)}^{2}}\;$$

By using Equation (9), we can calculate the RMSE value [23].

#### 3.2.10. Relative Absolute Error

Relative Absolute error is expressed as a ratio, and a minor error is compared to the errors produced by a small model. Mathematically, the RAE can be calculated by using the formula below:

(10) $$RAE=\frac{{\left[\sum_{i=1}^{n}{\left(P_{i}-A_{i}\right)}^{2}\right]}^{2}}{{\left[\sum_{i=1}^{n}A_{i}^{2}\right]}^{2}}$$

By using Equation (10), we can calculate the RAE value.

#### 3.2.11. Root Relative Absolute Error

RRAE (Relative Ratio Absolute Error) is a metric that can be used to evaluate the performance of a predictive model. It is calculated by dividing the Mean Absolute Error (MAE) of the model by the error of the Zero-R classifier, which is a simple classifier that always predicts the most frequent class in the training data. The formula for RRAE is

(11) $$RRAE=\sqrt{\frac{\sum_{j=1}^{n}{\left(P_{j}-T_{j}\right)}^{2}}{\sum_{j=1}^{n}{\left(T_{j}-T_{j}\right)}^{2}}}$$

By using Equation (11), we can calculate the RRAE value.

## 4. Methods and Results

This experiment uses two sets of programming tools in WEKA (Waikato Environment for Knowledge Analysis), a combination of machine learning algorithms utilizing Python libraries such as MATLAB, Jupiter Notebook, and Scikit Learn Library. The result is generated from both programming tools which compares their results and graphs and calculates accuracy, error rate, F-measure, PRC (precision–recall curves area), MCC (Matthew’s correlation coefficient), and ROC (receiver operating characteristic) areas. These experiments are performed using classification algorithms, for instance, Random Forest, Logistic Regression, K-Nearest Neighbors, Decision Tree, Naïve Bayes, and Support Vector Machine. The results and evolutions that we have performed and concluded, using both WEKA and Python, have been uploaded to GitHub, which is publicly accessible at https://github.com/wisalsafi/Diabetes-Prediction-Using-Machine-Learning-Algorithms-with-PIMA-Diabetes-Dataset/tree/main/ML%20Algoritms%20for%20Diabetes accessed on 7 July 2023.

The dataset used in the proposed work is the PIMA diabetes dataset, which consists of 768 instances. We performed our experiments and split the dataset by using a percentage split. “Percentage split” in machine learning often refers to dividing a dataset into separate subsets for training, validation, and testing. To assess the effectiveness of machine learning models and make sure they generalize successfully to new, unexplored data, segmentation is a crucial step. It is essential to choose a distribution that suits your particular situation, the size of your dataset, and the availability of the data. When the dataset is small, you can also use methods like cross-validation to obtain the most out of your data.

### 4.1. Decision Tree

The Decision Tree algorithm is utilized in this paper on the grounds that the Decision Tree calculation has the best precision proportion in the PIMA dataset and enjoys a few benefits, for example, it is not difficult to peruse and decipher, it is simple to get ready, and it requires less information cleaning. Decision Trees are regulated AI calculations that are utilized for both grouping and relapse strategies. Managed implies it requires a name for its execution. A Decision Tree is a supporting apparatus with a tree-like design that models potential results and assets. Decision Trees furnish a method for giving calculations contingent control articulations. They incorporate branches that address the dynamic advances that can prompt the endorsement of results [24]. The Decision Tree gives a solid strategy to arrangements and expectations in the analysis of diabetes. There are different types of Decision Trees available for classifying data, such as ID3, C4.5, C5.0, CART, Decision Stump, J48, and Heoffding Tree [19].

#### 4.1.1. Mathematical Equations

Decision Trees have three main mathematical formulas, including information gain, entropy, and gain.

##### Information Gain

Information gain is a measure that determines the change in entropy when a dataset is partitioned based on an attribute. It indicates the amount of new information that the attribute provides about the target variable. The Decision Tree construction process relies on information gain values to determine the sequence in which nodes should be split. The algorithm aims to maximize the information gain at each step, selecting the attribute that offers the greatest information gain as the first to be split. The value can be computed using the formula below:

(12) $$Info\left(samples\right)=-\overset{m}{\underset{i=1}{\sum }}pi\;log_{2}\left(pi\right)\;$$

From Equation (12), we can calculate information gain.

##### Entropy

Entropy is a metric to compute impurities in certain attributes. It determines randomness in the data. Entropy can be calculated as follows:

(13) $$E\left(T,\;X\right)=\overset{m}{\underset{i=1}{\sum }}p\left(c\right)E\left(c\right)\;$$

From Equation (13), we can calculate Entropy.

##### Gain

The gain is a measure of impurity or purity used when constructing a Decision Tree in the CART algorithm. Gain can be calculated using the formula below:

(14) Gain=E(samples)−E(T, X) 

From Equation (14), we can calculate Gain.

#### 4.1.2. Confusion Matrix

A confusion matrix is a table that summarizes the results of a classification model on a set of input data. It presents the count of correct and incorrect predictions for each class [5]. The matrix includes four categories:

- True Positive (TP): It is positive for both observed and predicted.
- False Positive (FP): It is negative for both observed and predicted.
- False Negative (FN): It is positive for observed, but negative for predicted.
- True Negative (TN): It is positive for predicted, but negative for observed.

To calculate the accuracy score, the correct values are placed along a diagonal line from the top left to the bottom right of the matrix. Adding the values on this diagonal line (134 + 48 = 182) gives the accuracy of the model, which, in this case, is 79.13%.

#### 4.1.3. Result through WEKA

Table 3 shows the confusion matrix by WEKA by applying the decision tree classification algorithm.

Table 4 shows actual class, ICI, and accuracy details, and Table 5 shows the confusion matrix [25].

India’s PIMA diabetes dataset uses 760 instances, but here we experimented on the WEKA tool by using a percentage split and then taking a percentage share of 70%, which holds only 230 instances, and performing experiments that give us the best accuracy ratio, like the FPR (false positive ratio), TPR (true positive ratio), Prec (precision), F-M (F-measure), and Rec (recall).

#### 4.1.4. Result through Python

Table 6 shows the confusion matrix by Python by applying the decision tree classification algorithm.

Table 7 shows actual class, ICI, and accuracy details, whereas Table 8 shows the confusion matrix [25].

### 4.2. Logistic Regression

Logistic Regression is a supervised machine learning algorithm that models the likelihood of discrete outcomes when input variables are given. This helps in understanding the alliance between multiple independent variables and one dependent variable [8]. It is an algorithm that takes two values, Yes or No, 0 or 1, and True or False [9]. Logistic Regression is a transformation of linear regression using the sigmoid function. Logistic Regression solves many problems encountered in fermium product development for which linear regression cannot be performed because, instead of predicting a numerical value (for example, a user’s total lifetime income), it predicts a discrete and dichotomous value (e.g., the user will spend or not spend money for the product). For this reason, Logistic Regression might more accurately be termed logistic classification [26].

#### 4.2.1. Linear Regression

(15) $$y=\beta_{0}+\beta_{1}X_{1}+\beta_{2}X_{2}+\cdots +\beta_{n}X_{0}$$

where

- *y* is the dependent variable.
- *β*_0_, *β*_1_, *β*_2_, …, *β_n_* are the regression coefficients for the intercept.
- *X*_1_, *X*_2_, *X*_3_, … and *X_n_* are the independent variables [26].

#### 4.2.2. Sigmoid Function


(16) $$p=\frac{1}{1+e^{-y}}\;$$

Apply the sigmoid function on linear regression and the equation will be

(17) $$p=\frac{1}{1+e^{\beta_{0}+\beta_{1}X_{1}+\beta_{2}X_{2}+\cdots +\beta_{n}X_{0}}}$$

where

- *p* is the predicted probability of the dependent variable being 1 [26].

#### 4.2.3. Result through WEKA

Table 9 shows the confusion matrix by WEKA by applying the logistic regression algorithm.

Table 10 show actual class, ICI, and accuracy details, and Table 11 show the confusion matrix [25].

#### 4.2.4. Result through Python

Table 11 shows the confusion matrix by Python by applying the logistic regression algorithm.

**Table 11:** Confusion matrix through Python using logistic regression algorithm.

		Actual Values	
**Predicted** **Values**		**Positive**	Negative
	**Positive**	**138 (TP)**	20 (TN)
	**Negative**	27 (FN)	**44 (FP)**

Table 12 shows actual class, ICI, and accuracy details [25].

### 4.3. Random Forest

Random Forest is a machine learning algorithm that has evolved from a Decision Tree [27]. Random Forest is used in classification models. It works in the following way: to classify an attribute, every Decision Tree algorithm comes up with a classification on the input data given, and afterward, Random Forest collects and predicts the most casted ballot expectation. Basically, the input from each tree is sample data from the dataset. In basic words, Random Forest uses many models of Decision Trees to achieve a better prediction model.

#### 4.3.1. Mathematical Equations

(18) $$F_{RF}\left(x\right)=\frac{1}{T}\;\overset{T}{\underset{t=1}{\sum }}F_{t}\left(x\right)\;$$

where:

- F_RF_ (x) is the prediction of Random Forest for input x.
- F_t_ (x) is the prediction of t-th Decision Tree for input x.
- T is the total number of Decision Trees in the Random Forest [27].

#### 4.3.2. Result through WEKA

Table 13 shows the confusion matrix by WEKA by applying the random forest algorithm.

Table 14 shows the details accuracy by utilizing WEKA [25].

#### 4.3.3. Result through Python

Table 15 shows the confusion matrix by Python by applying the random forest algorithm.

Table 16 shows the details accuracy by utilizing Python [25].

### 4.4. K-Nearest Neighbors

KNN is a classification-focused supervised machine learning algorithm [11]. K stands for the quantity of nearest neighbors in KNN. The K value affects how well this algorithm performs. This is a non-parametric algorithm, and learning is slow because it does not pick up during the training stage, is very simple to implement, and is applied on a large dataset [8]. KNN performs three fundamental stages: distance estimation, looking for adjacent neighbors, and deciding in favor of marks [15].

#### 4.4.1. Mathematical Equation


(19) $$D_{H}=\overset{k}{\underset{i=0}{\sum }}\left|x_{i}-y_{i}\right|$$

#### 4.4.2. Result through WEKA

Table 17 shows the confusion matrix by WEKA by applying the K-NN algorithm.

Table 18 shows actual class, ICI, and accuracy details, and Table 19 shows the confusion matrix [25].

#### 4.4.3. Result through Python

Table 20 shows the confusion matrix by Python by applying the K-NN algorithm [28].

Following Table 21 shows actual class, ICI, and accuracy details, and Table 22 shows the confusion matrix [25].

### 4.5. Naïve Bayes

The Naïve Bayes algorithm is a supervised machine learning algorithm. Naïve Bayes is based on the Bayesian theorem, which shows the probability of a hypothesis when the proof is provided. This algorithm also states that each feature of a particular dataset makes an equal and independent contribution to the problematic target class [5]. The Naïve Bayes algorithm shows good performance on huge datasets having high dimensionality [11].

#### 4.5.1. Mathematical Equation


(20) $$P(H|S)=\frac{P\left(S|H\right)P\left(H\right)}{P\left(S\right)}$$

- P(H): The probability that hypothesis H is correct. P(S): Probability of S data. Previous probability of H [11].
- P(H|S): Probability of hypothesis H given data D.
- P(S|H): The probability that the S data is given the hypothesis H.

#### 4.5.2. Result through WEKA

Table 23 shows the confusion matrix by WEKA by applying the Naïve Bayes algorithm.

Table 24 shows actual class, ICI, and accuracy details, and Table 25 shows the confusion matrix [25].

#### 4.5.3. Result through Python

Table 26 shows the confusion matrix by Python by applying the Naïve Bayes algorithm.

Table 27 shows actual class, ICI, and accuracy details, and Table 28 shows the confusion matrix [25].

### 4.6. Support Vector Machines

SVM is a supervised machine learning algorithm, which is used for both classification and regression problem types. It can easily perform multiple categorical and continuous variables [29]. SVM plays out the classification task by developing a hyperplane in a multi-faceted space that isolates instances of various class marks [30]. The hyperplane is a decision point that separates a set of objects that have different class labels. SVM has many applications, such as email classification, face detection, instruction detection, and gene classification.

#### 4.6.1. Mathematical Equation

(21) $$L_{p}=\frac{1}{2}\|\vec{W}\|-\overset{t}{\underset{i=1}{\sum }}\propto_{i}y_{i}\left(\vec{W}\cdot \vec{X}\right)+\overset{t}{\underset{i=1}{\sum }}\propto_{i}\;$$

where

- *t* is the number of prepared models.
- $\propto_{i}$, i = 1, …, t, are non-negative numbers.
- Derivatives of L P with respect to α*_i_* are zero.
- $\propto_{i}$ are the Lagrangian multipliers.
- $L_{p}$ is called Lagrangian.

The vector w and the constant b define the hyperplane [31].

#### 4.6.2. Result through WEKA

Table 29 shows the confusion matrix by WEKA by applying the Support Vector Machine algorithm.

Table 30 shows actual class, ICI, and accuracy details, and Table 31 shows the confusion matrix [25].

#### 4.6.3. Result through Python

Table 32 shows the confusion matrix by Pyton by applying the Support Vector Machine algorithm.

Table 33 shows actual class, ICI, and accuracy details, and Table 34 shows the confusion matrix [25].

### 4.7. Accuracy and Inconsistence Rate

**Figure 1:**
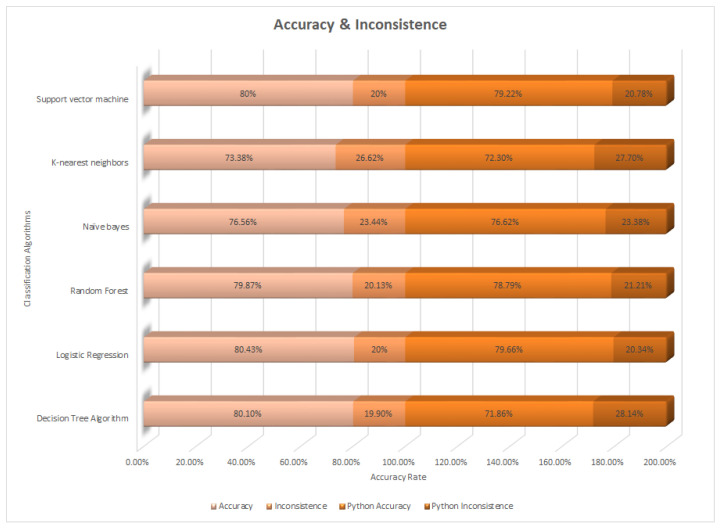
Accuracy and inconsistence rate.

Table 35, below, shows the accuracy (actual class) and inconsistence (incorrectly classified instances) by using the WEKA tool and the Python library. We used six classification algorithms with different accuracy rates and selected the algorithm on the basis of the highest accuracy rate. We performed the experiment on different classification algorithms, and the result is shown in Table 31, as well as in Figure 1. We conclude that the highest and best accuracy was obtained by Logistic Regression at 80.43%, and the second highest accuracy was obtained by Decision Tree and Support Vector Machine, with 79.13% accuracy. The third one was Naïve Bayes, with an accuracy of 76.96% obtained through WEKA. By using the Python library, we obtained an accuracy better than WEKA, which has the highest accuracy of 81% obtained by Logistic Regression.

### 4.8. MCC, ROC, and PRC Area

**Figure 2:**
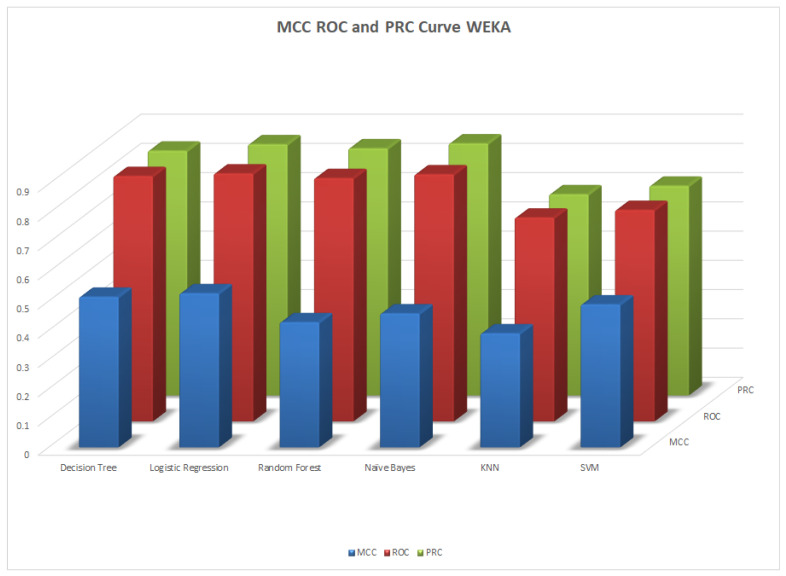
MCC, ROC, and PRC by WEKA.

**Figure 3:**
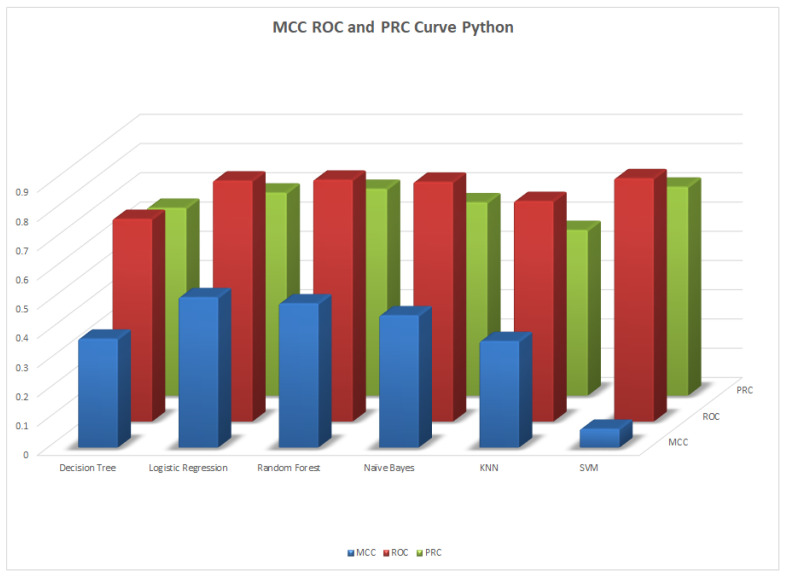
MCC, ROC, and PRC by Python.

We illustrate the values and graph of the MCC (Matthews correlation coefficient), which is between +1 and −1; the MCC perfect score is +1 and the worst score is −1 [32]. The ROC (receiver operating characteristics) graphical plot shows the diagnostic values between 0 and 1 [33]. PRC (precision–recall curve) should be used when there is a moderate to large class imbalance; all of these have different values between 0 and 1 [34]. A comparison between WEKA and Python results is shown in Table 36 along with Figure 2 and Figure 3.

### 4.9. Performance Metrics

**Figure 4:**
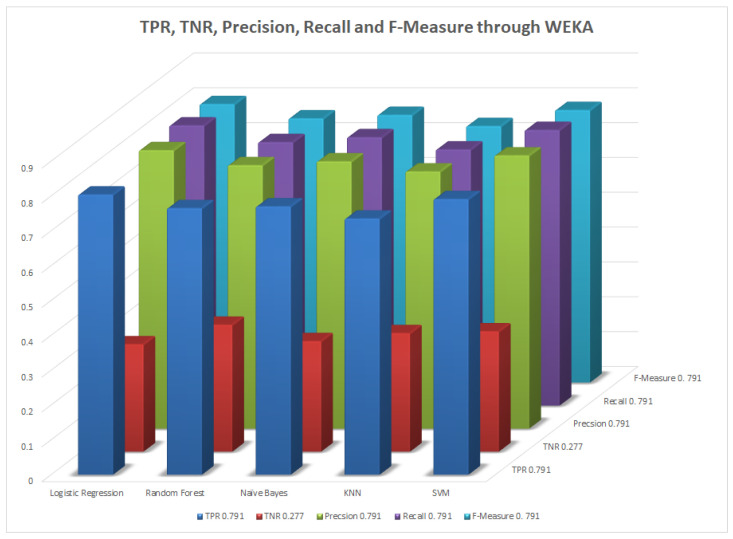
Performance metric through WEKA.

**Figure 5:**
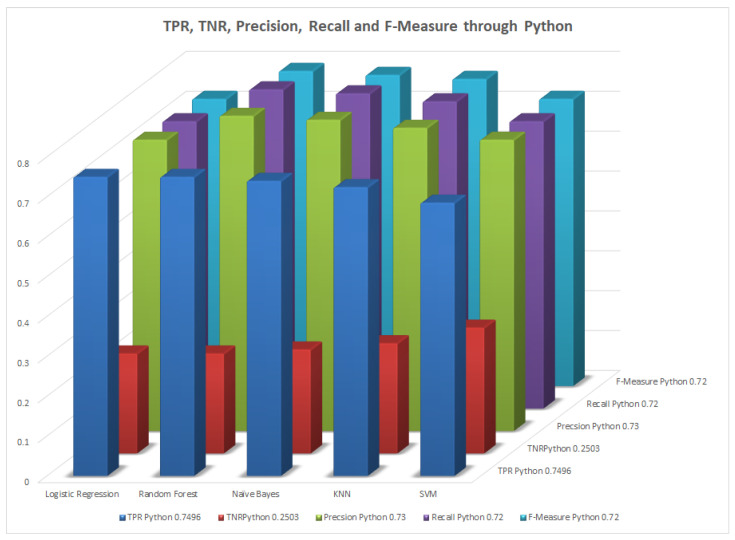
Performance metrics through Python.

**Figure 6:**
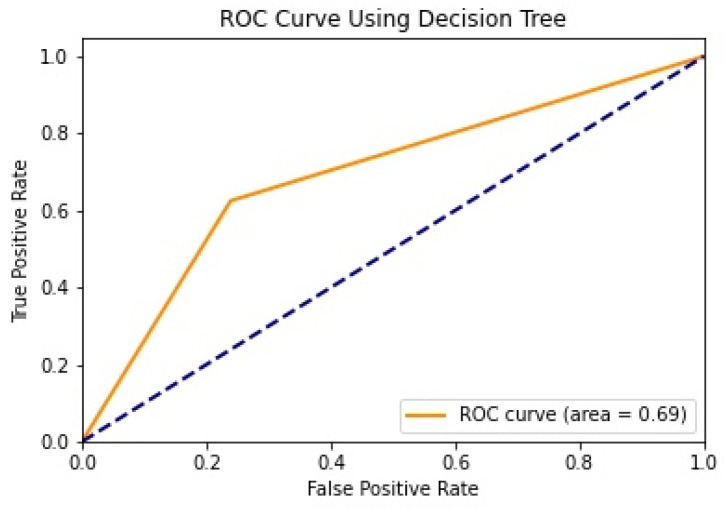
ROC curve using Decision Tree.

**Figure 7:**
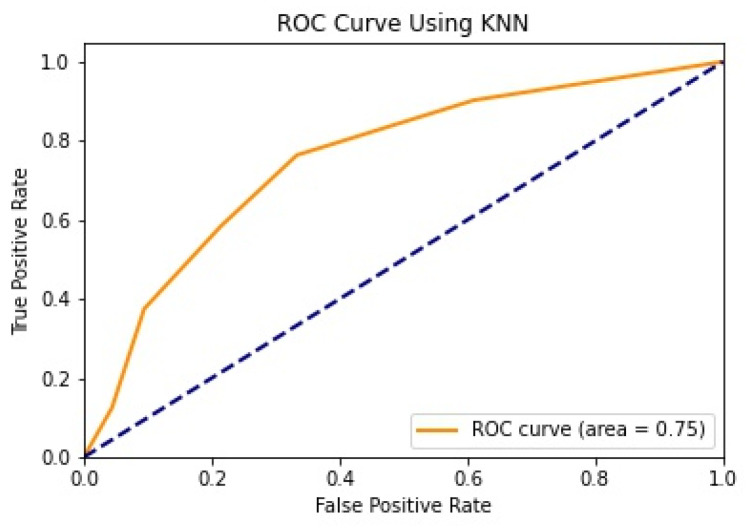
ROC curve using KNN.

**Figure 8:**
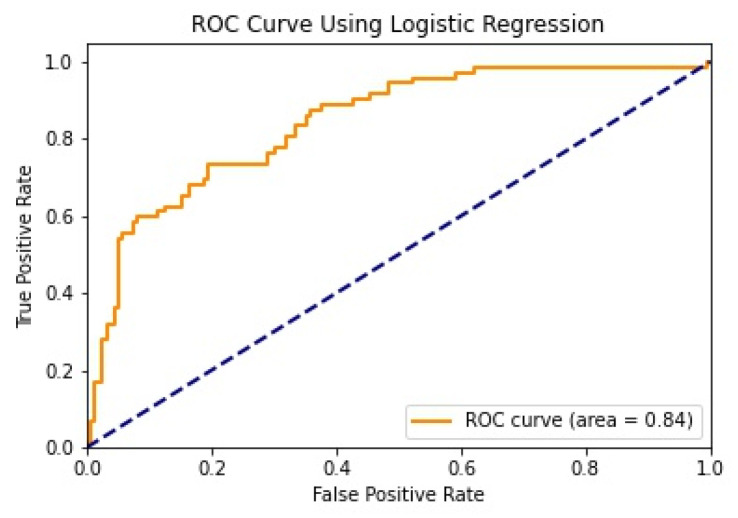
ROC curve using Logistic Regression.

**Figure 9:**
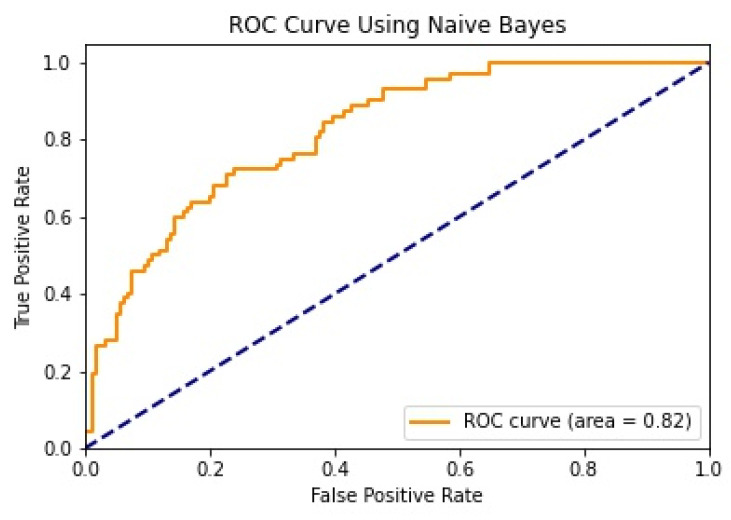
ROC curve using Naïve Bayes.

**Figure 10:**
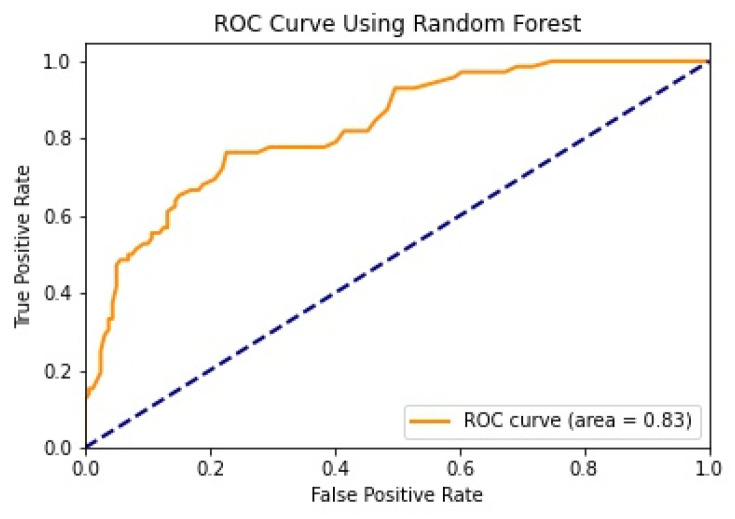
ROC curve using Random Forest.

**Figure 11:**
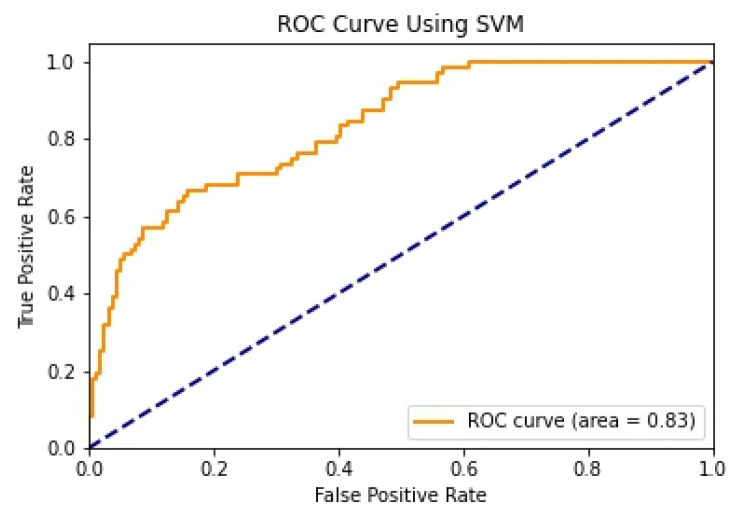
ROC curve using SVM.

We mentioned some important errors in our research paper, which are shown in the form of tables and graphs. The true positive rate (TPR) and the true negative rate (TNR) are performance metrics used in machine learning, especially in the context of binary classification problems [35]. They provide insight into the accuracy of model predictions for positive and negative samples. An important metric used to evaluate exactness is precision. Recall shows what percentage of occurrences were really categorized as belonging to a particular class. The F-measure score, which is the weighted average (harmonic mean) of the accuracy and memory results, may be obtained by combining the two metrics of precision and recall, and it was used to predict the sample [13]. The following performance metrics are shown in Table 37 and Table 38 along with Figure 4, Figure 5, Figure 6, Figure 7, Figure 8, Figure 9, Figure 10 and Figure 11 respectively along with ROC curve graphs of all implemented classification algorithms.

## 5. Conclusions

Diabetes is a complex medical disease that affects how the body uses glucose, a form of sugar that is the body’s primary fuel source. Diabetes results when our body is unable to make insulin or is unable to use the insulin produced by our body. According to 2017 statistics, 425 million people worldwide are suffering from diabetes. Diabetes causes 2 to 5 million deaths worldwide each year. By 2045, this is expected to increase to 629 million [36]. Type 1 diabetes, Type 2 diabetes, and gestational diabetes are the three main subtypes of diabetes. In this paper, we studied and tried to predict diabetes using six different classification algorithms applied to the PIMA diabetes dataset. We implemented and evaluated the results. The toolsets we used were WEKA, Python libraries like MATLAB, and Juypter Notebook. The main objective of our research work was to predict diabetes and select the algorithm that has the highest accuracy, such as Logistic Regression (81%), Decision Tree (80.10%), and SVM (80%). It has more than one calculated error, accuracy, precision, and F-measure. Finally, the study findings have important implications and offer benefits to various groups, including patients, healthcare providers, public health agencies, researchers, and insurance companies. The use of web-based diabetes prediction tools has the potential to provide timely support and treatment to at-risk individuals, leading to improved health outcomes and reduced healthcare costs. In the future, we aim to develop a complete web-based application with a medical module, user notification, and live chat box, and also aim to provide an Android application. We will also investigate the performance of the system and train some better accuracy algorithms.

## Figures and Tables

**Table 1:** Comparison to past literature.

Author	Dataset	Technique	Tools	Accuracy
Deepti Sisodia et al. [5]	PIDD	SVM, Naïve Bayes, Decision Tree	WEKA	76.30%
Steffi Dr. R. Balsubram et al. [6]	PIDD	SVM, Decision Tree, Decision Table	MATLAB	74.9%
R. Madhusmita, Amandeep, K et al. [8]	PIDD	LR, SVM, KNN, NB, DT	-	82.35%
Varma, K. M., and Panda, B. S et al. [9]	PIMA	Naïve Bayes, SVM, Logistic Regression, Decision Tree	R-tool	74.67%
Wu, H., Yang, S., Huang, Z., He, J., and Wang, X. et al. [10]	PIMA	K-means, Logistic Regression	WEKA	Applicable
O. Dr. O., S. Dr. K., and B Ramudu et al. (2020) [11]	PIMA	Clustering regression, SVM, KNN, Neural Network	R-Studio	78%
Talha Mahboob Alama, Muhammad Atif Iqbala et al. [15]	UCI ML Repository	ANN, K-Mean, Random Forest	-	75.7%
Mukesh kumari1, Dr. Rajan Vohra, Anshul arora et al. [16]	PIMA	Bayesian Network Classifier	WEKA	70.60%
Mir, A., and Dhage, S. N. et al. [17]	PIMA	Naïve Bayes, SVM, CART, Random Forest	WEKA	79.13%
Huma. Naz and Sachin. Ahuja et al. [18]	PIMA	ANN, DT, DL, NB	-	90–98%
A. Iyer, J. S, and R. Sumbaly et al. [19]	PIMA Dataset	DT, NB	WEKA	79%
Abdulhadi, N., and Al-mousa, A. et al. [13]	PIMA Dataset	RF, SVM, LDA, VC, SCV	Python	82%
Chou, C.-Y.; Hsu, D.-Y.; Chou, C.-H. et al. [14]	PIMA Dataset with Patients ID	LR, Bi-NN, RF, DT	Python	95%

**Table 2:** Attribute descriptions.

Attribute Name	Attribute Description	Min	Max	Mean	St_dev
Pregnancies	Number of times pregnant	0.00	17.00	3.845	3.37
Glucose	2 h plasma glucose concentration in an oral glucose tolerance test	0.00	199.00	120.895	31.973
Blood Pressure	Diastolic blood pressure (mm Hg)	0.00	122.00	69.105	19.356
Skin Thickness	Triceps skin fold thickness (mm)	0.00	99.00	20.536	15.244
Insulin	Serum insulin 2 h (muU/mL)	0.00	846	79.799	115.244
BMI	Body mass index (weight in kg/(height in m)^2^)	0.00	67.10	31.993	7.884
Diabetes Pedigree Function	Diabetes pedigree function	0.00	2.24	0.472	0.331
Age	Age (year)	0.00	81.00	33.241	11.78
Class	Class variable (1 for positive and 0 for negative)	0.00	1.00	-	-

**Table 3:** Confusion matrix through WEKA using the decision tree algorithm.

		Actual Values	
**Predicted** **Values**		**Positive**	Negative
	**Positive**	**134 (TP)**	24 (TN)
	**Negative**	24 (FN)	**48 (FP)**

**Table 4:** Actual class and ICI details achieved via Decision Tree through WEKA.

Decision Tree	No. of Instances
Actual Class	182

**Table 5:** Decision Tree details of accuracy through WEKA.

Class	TPR	FPR	Prec	Rec	F-M
0	0.848	0.333	0.848	0.848	0.848
1	0.667	0.152	0.667	0.667	0.667
Weighted Average	0.791	0.277	0.791	0.791	0.791

**Table 6:** Confusion matrix through Python using decision tree algorithm.

		Actual Values	
**Predicted** **Values**		**Positive**	Negative
	**Positive**	**120 (TP)**	38 (TN)
	**Negative**	27 (FN)	**45 (FP)**

**Table 7:** Actual class and ICI details achieved via Decision Tree through Python.

Decision Tree	No. of Instances
Actual Class	165
Incorrectly Classified Instances	65
Total Number of Instances	230

**Table 8:** Decision Tree details of accuracy through Python.

Class	TPR	FPR	Prec	Rec	F-M
0	0.625	0.1257	0.82	0.76	0.79
1	0.8742	0.375	0.54	0.62	0.58
Weighted Average	0.7496	0.2503	0.73	0.72	0.72

**Table 9:** Confusion matrix through WEKA using logistic regression algorithm.

		Actual Values	
**Predicted** **Values**		**Positive**	Negative
	**Positive**	**142 (TP)**	16 (TN)
	**Negative**	29 (FN)	**43 (FP)**

**Table 10:** Actual class and ICI details achieved via Logistic Regression through WEKA.

Logistic Regression	No. of Instances
Actual Class	185
Incorrectly Classified Instances	45
Total Number of Instances	230

**Table 12:** Actual class and ICI details achieved via Logistic Regression through Python.

Logistic Regression	No. of Instances
Actual Class	183
Incorrectly Classified Instances	47
Total Number of Instances	230

**Table 13:** Confusion matrix through WEKA using random forest algorithm.

		Actual Values	
**Predicted** **Values**		**Positive**	Negative
	**Positive**	**138 (TP)**	20 (TN)
	**Negative**	34 (FN)	**38 (FP)**

**Table 14:** Random Forest details of accuracy through WEKA.

Class	TPR	FPR	Prec	Rec	F-M
0	0.873	0.472	0.802	0.873	0.836
1	0.528	0.127	0.655	0.528	0.585
Weighted Average	0.765	0.364	0.756	0.756	0.758

**Table 15:** Confusion matrix through Python using random forest algorithm.

		Actual Values	
**Predicted** **Values**		**Positive**	Negative
	**Positive**	**137 (TP)**	21 (TN)
	**Negative**	28 (FN)	**45 (FP)**

**Table 16:** Random Forest details of accuracy through Python.

Class	TPR	FPR	Prec	Rec	F-M
0	0.6111	0.1320	0.83	0.87	0.85
1	0.8679	0.3888	0.68	0.61	0.64
Weighted Average	0.7395	0.2604	0.78	0.79	0.78

**Table 17:** Confusion matrix through WEKA. using K-NN algorithm.

		Actual Values	
**Predicted** **Values**		**Positive**	Negative
	**Positive**	**126 (TP)**	32 (TN)
	**Negative**	29 (FN)	**43 (FP)**

**Table 18:** Actual class and ICI details achieved via KNN through WEKA.

KNN	No. of Instances
Actual Class	169
Incorrectly Classified Instances	61
Total Number of Instances	230

**Table 19:** KNN details of accuracy through WEKA.

Class	TPR	FPR	Prec	Rec	F-M
0	0.797	0.403	0.813	0.797	0.805
1	0.597	0.203	0.573	0.597	0.585
Weighted Average	0.735	0.340	0.738	0.735	0.736

**Table 20:** Confusion matrix through Python.

		Actual Values	
**Predicted** **Values**		**Positive**	Negative
	**Positive**	**124 (TP)**	34 (TN)
	**Negative**	30 (FN)	**42 (FP)**

**Table 21:** Actual class and ICI details achieved via KNN through Python.

Naïve Bayes	No. of Instances
Actual Class	166
Incorrectly Classify Instances	64
Total Number Instances	230

**Table 22:** KNN details of accuracy through Python.

Class	TPR	FPR	Prec	Rec	F-M
0	0.5833	0.2138	0.81	0.79	0.80
1	0.7861	0.4166	0.55	0.58	0.57
Weighted Average	0.6847	0.3152	0.73	0.72	0.72

**Table 23:** Confusion matrix through WEKA using Naïve Bayes Algorithm.

		Actual Values	
**Predicted** **Values**		**Positive**	Negative
	**Positive**	**133 (TP)**	25 (TN)
	**Negative**	28 (FN)	**42 (FP)**

**Table 24:** Actual class and ICI details achieved via Naïve Bayes through WEKA.

Naïve Bayes	No. of Instances
Actual Class	177
Incorrectly Classified Instances	53
Total Number of Instances	230

**Table 25:** Naïve Bayes details of accuracy through WEKA.

Class	TPR	FPR	Prec	Rec	F-M
0	0.842	0.389	0.826	0.842	0.834
1	0.611	0.158	0.638	0.611	0.624
Weighted Average	0.770	0.317	0.767	0.770	0.768

**Table 26:** Confusion matrix through Python using Naïve Bayes algorithm.

		Actual Values	
**Predicted** **Values**		**Positive**	Negative
	**Positive**	**132 (TP)**	26 (TN)
	**Negative**	28 (FN)	**44 (FP)**

**Table 27:** Actual class and ICI details achieved via Naïve Bayes through Python.

Naïve Bayes	No. of Instances
Actual Class	176
Incorrectly Classified Instances	54
Total Number of Instances	230

**Table 28:** Naïve Bayes details of accuracy through Python.

Class	TPR	FPR	Prec	Rec	F-M
0	0.6111	0.1635	0.83	0.84	0.83
1	0.8364	0.3888	0.63	0.61	0.62
Weighted Average	0.7237	0.2762	0.76	0.77	0.77

**Table 29:** Confusion matrix through WEKA.

		Actual Values	
**Predicted** **Values**		**Positive**	Negative
	**Positive**	**143 (TP)**	15 (TN)
	**Negative**	33 (FN)	**39 (FP)**

**Table 30:** Actual class and ICI details achieved via SVM through WEKA.

SVM	No. of Instances
Actual Class	182
Incorrectly Classify Instances	48
Total Number Instances	230

**Table 31:** SVM details of accuracy through WEKA.

Class	TPR	FPR	Prec	Rec	F-M
0	0.905	0.458	0.813	0.705	0.856
1	0.542	0.095	0.722	0.542	0.619
Weighted Average	0.791	0.345	0.784	0.791	0.782

**Table 32:** Confusion matrix through Python using Support Vector Machine algorithm.

		Actual Values	
**Predicted** **Values**		**Positive**	Negative
	**Positive**	**13 (TP)**	20 (TN)
	**Negative**	28 (FN)	**44 (FP)**

**Table 33:** Actual class and ICI details achieved via SVM through Python.

SVM	No. of Instances
Actual Class	182
Incorrectly Classify Instances	48
Total Number Instances	230

**Table 34:** SVM details of accuracy through Python.

Class	TPR	FPR	Prec	Rec	F-M
0	0.5604	0.1079	0.83	0.87	0.85
1	0.8930	0.4305	0.69	0.61	0.65
Weighted Average	0.7312	0.2687	0.79	0.79	0.79

**Table 35:** Accuracy and inconsistence rate.

Accuracy of Classification Algorithms	WEKA Accuracy	WEKA Inconsistence	Python Accuracy	Python Inconsistence
Decision Tree	80.10%	19.90%	71.86%	28.14%
Logistic Regression	80.43%	19.57%	79.66%	20.34%
Random Forest	79.87%	20.13%	78.79%	21.21%
Naïve Bayes	76.56%	23.44%	76.62%	23.38%
KNN	73.38%	26.62%	72.30%	27.70 %
SVM	80%	20%	79.22%	20.78%

**Table 36:** MCC, ROC, and PRA area.

Machine Learning Algorithms	MCC	ROC	PRC	Python MCC	Python ROC	Python PRC
Decision Tree	0.515	0.839	0.837	0.372	0.693	0.642
Logistic Regression	0.527	0.848	0.859	0.514	0.842	0.694
Random Forest	0.428	0.832	0.845	0.493	0.827	0.707
Naïve Bayes	0.458	0.845	0.862	0.451	0.820	0.661
KNN	0.39	0.697	0.688	0.364	0.754	0.566
SVM	0.489	0.723	0.717	0.064	0.832	0.714

**Table 37:** TPR, TNR, precision, recall, and F-measure through WEKA.

Algorithms	TPR	TNR	Precision	Recall	F-Measure
DT	0.791	0.277	0.791	0.791	0.791
LR	0.804	0.308	0.799	0.804	0.799
RF	0.765	0.364	0.756	0.756	0.758
NB	0.770	0.317	0.767	0.770	0.768
KNN	0.735	0.340	0.738	0.735	0.736
SVM	0.791	0.345	0.784	0.791	0.782

**Table 38:** TPR, TNR, precision, recall, and F-measure through Python.

Algorithms	TPR Python	TNR Python	Precision Python	Recall Python	F-Measure Python
DT	0.7496	0.2503	0.73	0.72	0.72
LR	0.7496	0.2503	0.79	0.80	0.79
RF	0.7395	0.2604	0.78	0.79	0.78
NB	0.7237	0.2762	0.76	0.77	0.77
KNN	0.6847	0.3152	0.73	0.72	0.72
SVM	0.7312	0.2687	0.79	0.79	0.79

## Data Availability

Not applicable.
